# Exploring optoelectronic and photocatalytic properties of X_2_AgBiY_6_ (X = NH_4_, PH_4_, AsH_4_, SbH_4_ and Y = Cl, Br): a DFT study

**DOI:** 10.1039/d3ra07460a

**Published:** 2024-01-19

**Authors:** Sardar Mohsin Ali, M. Usman Saeed, Hosam O. Elansary, Y. Saeed

**Affiliations:** a Department of Physics, Abbottabad University of Science and Technology Abbottabad KPK Pakistan yasi.saeed@kaust.edu.sa yasir.saeed@kaust.edu.sa yasirsaeedphy@aust.edu.pk +(92)-3454041865; b Prince Sultan Bin Abdulaziz International Prize for Water Chair, Prince Sultan Institute for Environmental, Water and Desert Research, King Saud University Riyadh 11451 Saudi Arabia; c Department of Plant Production, College of Food Agriculture Sciences, King Saud University Riyadh 11451 Saudi Arabia

## Abstract

*Ab initio* calculations have been used to investigate lead-free double-perovskites (DPs) X_2_AgBiY_6_ (X = NH_4_, PH_4_, AsH_4_, SbH_4_ and Y = Cl, Br) for solar-cell-based energy sources. The most recent and improved Becke–Johnson potential (TB–mBJ) has been proposed for the computation of optoelectronic properties. Theoretical and calculated values of the lattice constants obtained by applying the Wu–Cohen generalized gradient approximation (WC-GGA) were found to be in good agreement. The computed bandgap values of (NH_4_)_2_AgBiBr_6_ (1.574 eV) and (SbH_4_)_2_AgBiBr_6_ (1.440 eV) revealed their indirect character, demonstrating that they are suitable contenders for visible light solar-cell (SC) technology. Properties like the refractive index, light absorption, reflection, and dielectric constant are all explained in terms of the optical ranges. Within the wavelength range of 620–310 nm, the maximum absorption band has been identified. Additionally, we discover that all chemicals investigated herein have photocatalytic capabilities that can be used to efficiently produce hydrogen at cheap cost using solar water splitting by photocatalysts. In addition, the stability of the compounds was examined using the calculation of mechanical properties.

## Abstract 2

*Ab initio* calculations have been used to investigate lead-free double-perovskites (DPs) X_2_AgBiY_6_ (X = NH_4_, PH_4_, AsH_4_, SbH_4_ and Y = Cl, Br) for solar-cell-based energy sources.

## Graphical abstract

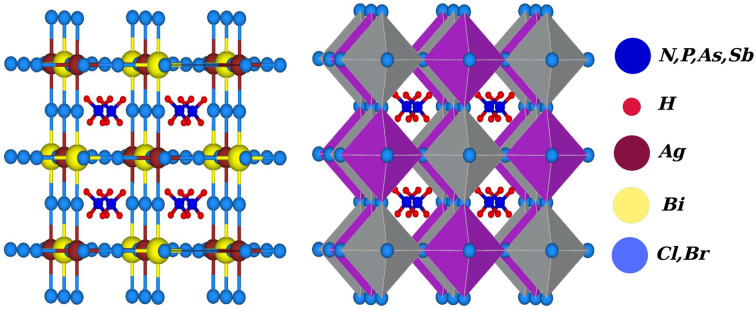

## 1. Introduction

**Fig. 1:**
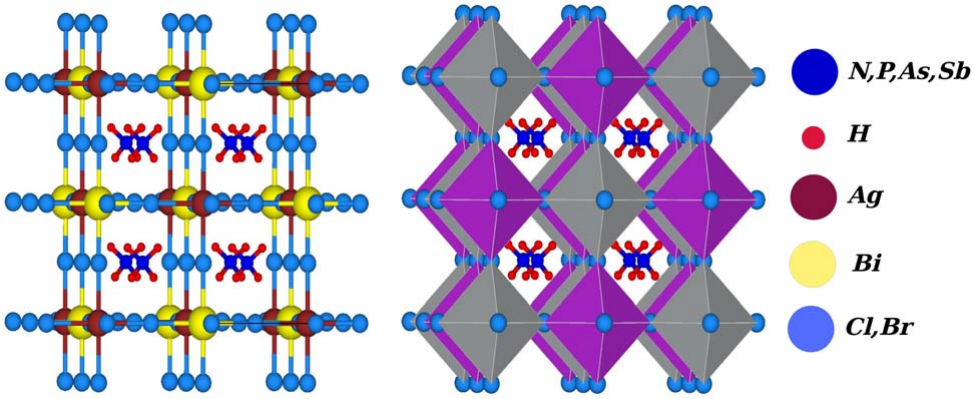
Predicted crystal structure of X_2_AgBiY_6_ (X = NH_4_, PH_4_, AsH_4_, SbH_4_ and Y = Cl, Br).

Due to the development of high efficiency and inexpensive materials, the past two decades have been crucial for the study of perovskite solar cells (PSC). Power conversion efficiencies (PCEs) for perovskite solar cells of up to 24.5% have been reported, which are comparable with PCEs for silicon (Si)-based solar cells.^1,2^ Recent perovskite solar cells function similarly to absorber materials made of an inorganic halide perovskite (HP) and a lead-based organic halide. Geisz *et al.*^3^ produced a multi-junction solar cell (SC) with a productivity greater than 47.1%, which is 11.1% higher than with thin-film-based solar cells. In extremely efficient solar cell applications, including the production of solar fuel and solar hydrogen, hybrid organic–inorganic halide perovskites CH_3_NH_3_PbX_3_ (X = Cl, Br, and I) have gained attention.^4–6^ On the other hand, lead-based halide perovskites have challenges with stability, as well as containing a hazardous material (lead), which limits the applications of these materials on a broad scale. Finding reliable and Pb-free alternative materials is therefore necessary to solve these issues. Therefore, halide double perovskites have received a lot of attention as a result.^7–17^ Halide double perovskites are a vast class of quaternary halides. Opportunities for new solar and photovoltaic materials have been offered by the wide geometric range of these materials. However, when a large number of compounds are examined to determine which are most promising in terms of energy, structure, and electrical properties for solar cell applications, the work grows more challenging. As opposed to the unstable solar absorbent substance CH_3_NH_3_PbI_3_ (MAPI), which contains Pb, halide double perovskite compounds like Cs_2_AgBiX_6_ (X = Cl and Br) have gained a lot of interest, leading to a number of experimental and theoretical studies.^18,19^ Filip *et al.*^20,21^ suggested other cation possibilities from hypothetical perovskites such as NH_4_, PH_4_, AsH_4_, SbH_4_, NCl_4_, PF_4_, Ch_3_PH_3_, *etc.* The thermodynamic stability of numerous halide double perovskites was predicted by Han and his colleagues using density functional theory (DFT).^22^ All-double perovskite inorganic materials have been investigated as a potential replacement for lead-based perovskites due to their non-toxic qualities and three-dimensional structures. Giustino and Snaith have predicted the formation of new halide double perovskites.^17^ From those suggested materials, we selected one class as X_2_AgBiY_6_ (X = NH_4_, PH_4_, AsH_4_, SbH_4_ and Y = Cl, Br) with the exception of F and I because of the corresponding substantial bandgap instability. A predicted view of the crystal structure of the stable double perovskite material X_2_AgBiY_6_ is illustrated in Fig. 1. In this paper, we investigated the structural, electronic, photocatalytic, optical, and mechanical characteristics of X_2_AgBiY_6_X_2_AgBiY_6_.

## 2. Computational details

We used the WIEN2k program to carry out our computations using the full-potential linearized augmented plane wave plus local orbitals (FP-LAPW + lo) approach, which constitutes a component of DFT.^23^ A more accurate exchange-correlation potential flavour can be acquired using the Wu–Cohen generalized gradient approximation (WC-GGA), and used to evaluate the properties of the material.^24^ In order to determine an accurate bandgap, we used the WC-GGA with the modified Becke–Johnson (mBJ) correction.^25,26^ For other semiconductors, especially lead halide perovskites, in the past,^27–31^ the mBJ approach produced encouraging band topologies and bandgap values when compared to experimental results. In order to apply the mBJ strategy across WC-GGA to our hypothesized compounds, we first applied it to the experimental data for Cs_2_AgBiX_6_ (X = Cl and Br), and after that, we implemented this plan for our compounds X_2_AgBiY_6_. Electronic and optical characteristics are estimated using *k*-meshes of 8 × 8 × 8 and 15 × 15 × 15. All structures are optimized when the energy convergence tolerance is 10^−5^ Ry.

## 3. Results and discussion

The structural characteristics of a compound play a significant role in understanding its varied physical properties. All eight potential compounds for the double perovskite X_2_AgBiY_6_ (X = NH_4_, PH_4_, AsH_4_, SbH_4_ and Y = Cl, Br) that we predicted are grouped into four different categories: NH_4_-, PH_4_-, AsH_4_-, and SbH_4_-based compounds.

**Fig. 2:**
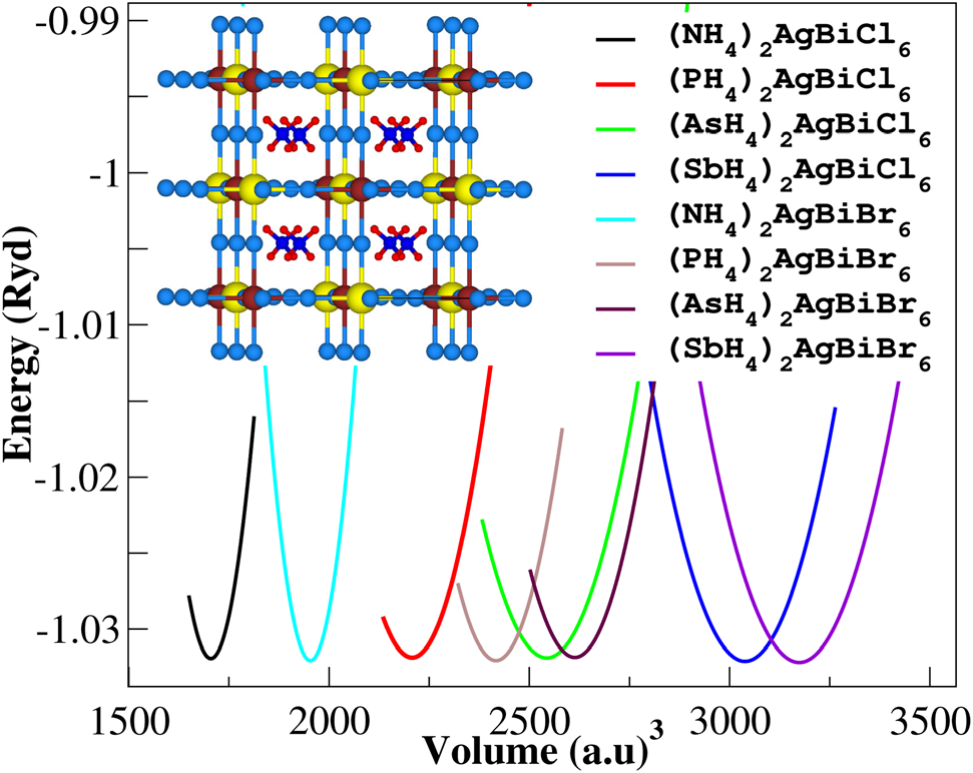
Volume optimization curves of X_2_AgBiY_6_.

Firstly, we performed relaxation as the organic components contain hydrogen. Then, using volume optimization, we were able to extract structural parameters such as the bulk modulus *B* (GPa) and lattice constant *a* (Å). Experimental values of the Cs_2_AgBiX_6_ lattice parameter (X = Cl and Br) has been used as a starting point. Table 1 displays the structural characteristics of the hypothetical compounds as determined using the Birch–Murnaghan equation of states.^32^ The optimization of X_2_AgBiY_6_ is presented in Fig. 2. The optimized lattice constants for all compounds range from 10.03 Å to 12.33 Å, which is quite close to the lattice constants of other experimentally synthesized double perovskites, like Cs_2_AgBiCl_6_ and Cs_2_AgBiBr_6_, which are 10.77 Å and 11.27 Å, respectively.^18^ (NH_4_)_2_AgBiCl_6_ has the greatest optimal bulk modulus, *B*, of all the compounds with a value of 75.55 GPa. (Sb_4_)_2_AgBiBr_6_, on the other hand, has the lowest *B* value of 27.15 GPa, demonstrating the durability of these double perovskites. Results for these optimized compounds cannot be compared to any theoretical or experimental data. After adjusting the lattice constant, these compounds can be characterized using bandgap values that are comparable to the best hybrid organic–inorganic perovskites.

**Table 1:** Lattice parameters, bond distances between A and H (A = N, P, As, and Sb) and bandgaps of halide double perovskites X_2_AgBiY_6_ (X = NH_4_, PH_4_, AsH_4_, SbH_4_ and Y = Cl, Br)

Materials	Bond distance *d*_(A–H)_ (Å)	Lattice parameter (Å)	Bulk modulus *B* (GPa)	Bandgap (without SOC) *E*_g_ (eV)	Bandgap (with SOC) *E*_g_ (eV)	Bandgap hybrid (mBJ + SOC) *E*_g_ (eV)
(NH_4_)_2_AgBiCl_6_	1.08	10.03	75.55	1.51	1.36	2.32
(NH_4_)_2_AgBiBr_6_	1.12	10.50	66.87	0.97	0.87	1.75
(PH_4_)_2_AgBiCl_6_	1.39	10.94	46.77	1.78	1.43	2.42
(PH_4_)_2_AgBiBr_6_	1.43	11.26	44.39	1.28	1.03	1.95
(AsH_4_)_2_AgBiCl_6_	1.45	11.47	36.41	1.83	1.52	2.29
(AsH_4_)_2_AgBiBr_6_	1.46	11.56	38.95	1.36	1.23	2.15
(SbH_4_)_2_AgBiCl_6_	1.66	12.15	26.18	1.80	1.44	2.00
(SbH_4_)_2_AgBiBr_6_	1.69	12.32	27.15	1.31	1.03	1.44

**Fig. 3:**
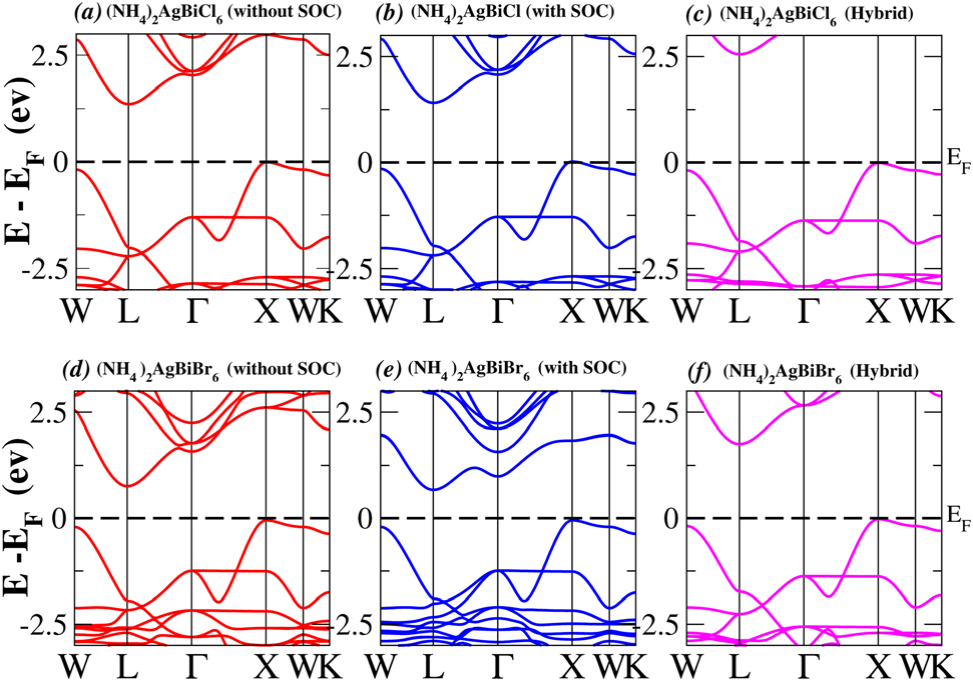
Band structure of (NH_4_)_2_AgBiY_6_.

**Fig. 4:**
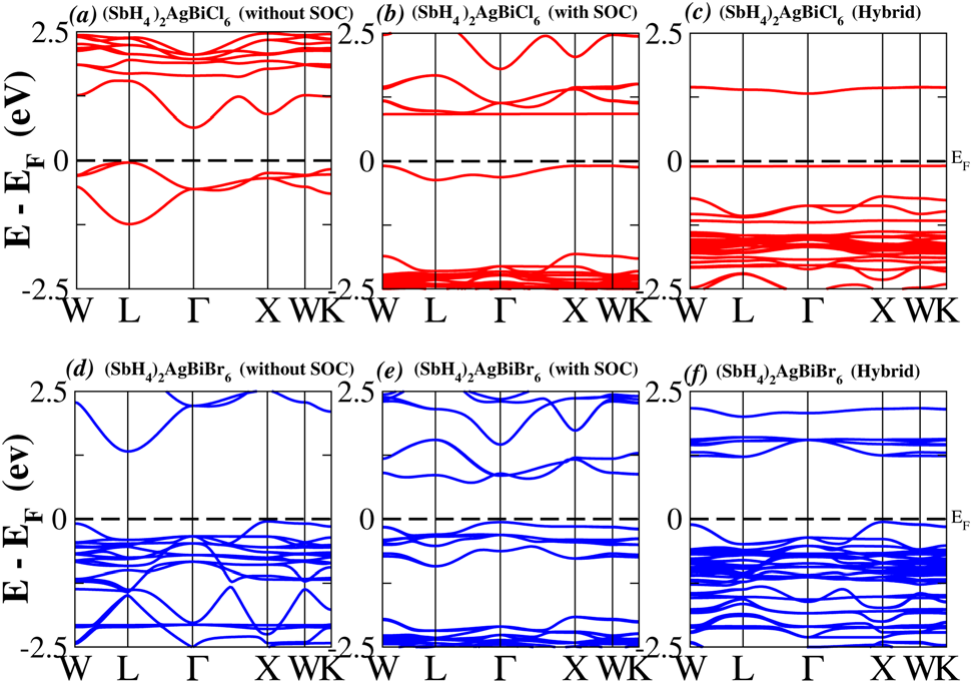
Band structure of (SbH_4_)_2_AgBiY_6_.

In order to analyse the electrical behavior of the materials, the band structures are essential. We can study the materials’ electronic nature by using band structures to determine if they are metallic or semiconducting, *etc.* The electronic band structure may successfully clarify the physical characteristics of solids including their optical behavior and resistivity, and this knowledge can further assist in the design of solid-state devices like solar cells and transistors, among other applications. In order to comprehend their electronic structure, we calculated the band structures of X_2_AgBiY_6_ (X = NH_4_, PH_4_, AsH_4_, SbH_4_ and Y = Cl, Br). We have observed a definite increase in their bandgap when their bond distance increases going from N to Sb. This is mostly caused by the compound’s growing number of deep shells. All of the compounds’ bandgaps were computed using an intuitive self-consistent field (SCF) model that took into account spin–orbit coupling (SOC), and on top of that, we used the mBJ technique to adjust the bandgap value to correspond with the experimental values. As a benchmark, we first recalculated the experimental bandgap values for Cs_2_AgBiCl_6_ and Cs_2_AgBiBr_6_, which are 2.77 eV and 2.19 eV, respectively.^18^ We have determined the bandgaps for Cs_2_AgBiBr_6_ to be 1.16 eV without SOC, 0.961 eV with SOC, and 2.17 eV with mBJ + SOC. For Cs_2_AgBiCl_6_, however, the bandgap values are 2.63 eV with mBJ + SOC, 1.40 eV with SOC, and 1.69 eV without SOC. For the first-Brillouin-zone results depicted in Fig. 3 and 4, the X_2_AgBiY_6_ electronic band structures were investigated at a high-symmetry point (X = Cl, Br). The band structures revealed an indirect bandgap for the DPs that included the conduction band’s L minima and valence band’s X and maxima at the L–X equilibrium point. On both a theoretical and practical level, lead-free DPs exhibit an indirect bandgap. Since we recently determined the bandgaps for (NH_4_)_2_AgBiBr_6_ (1.58 eV) and (SbH_4_)_2_AgBiBr_6_ by using mBJ + SOC to achieve bandgap values near to the MAPI experimental value, we were able to acquire indirect bandgaps comparable to MAPI.^33^ Therefore, we draw the conclusion that the FP-LAPW + lo approach significantly underestimates bandgap values in the double perovskites until the addition of mBJ + SOC. In order to replace MAPI at room temperature, we must therefore find a material that is lead-free and has a bandgap value that is almost comparable to 1.6 eV.^34^Table 1 provides calculated bandgap values calculated with and without SOC, and with mBJ and SOC, for all materials. Referring to Table 1, it becomes evident that only (NH_4_)_2_AgBiBr_6_ (1.58 eV) and (SbH_4_)_2_AgBiBr_6_ have small bandgap values of 1.75 eV and 1.44 eV, respectively, for mBJ + SOC. On the other hand, the bandgap values for the rest of the compounds exceed 1.80 eV, as shown in Table 1. These materials exhibit a very high bandgap when mBJ + SOC is applied, rendering them inappropriate for solar cell applications, although they are fit for photocatalytic applications.

**Fig. 5:**
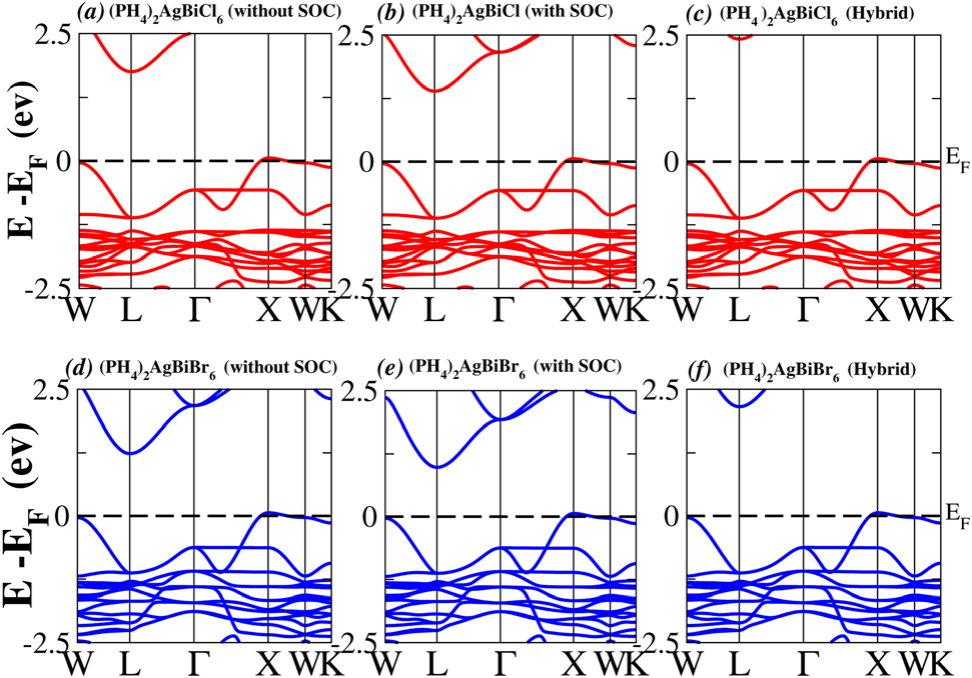
Band structure of (PH_4_)_2_AgBiY_6_.

**Fig. 6:**
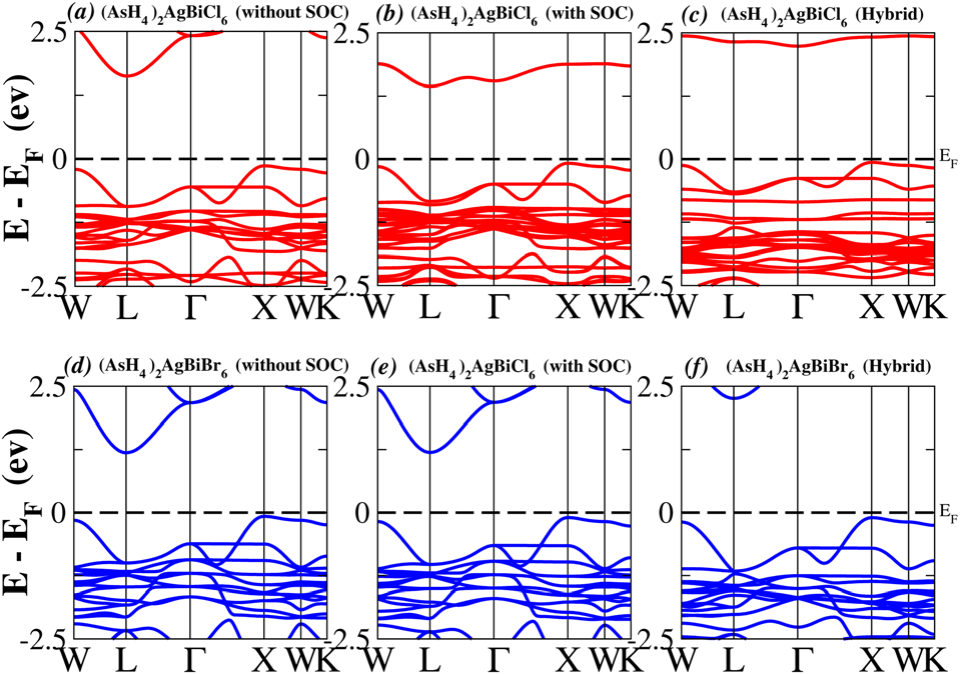
Band structure of (AsH_4_)_2_AgBiY_6_.

We determined bandgap values for X_2_AgBiY_6_ materials without SOC, with SOC, and with the hybrid calculation, as shown in Table 1. According to Fig. 3(a) and (b), and 4(a) and (b), (NH_4_)_2_AgBiBr_6_’s bandgap value is 0.97 eV without SOC, and 0.88 eV with SOC, and similarly for (SbH_4_)_2_AgBiBr_6_, the bandgap value is 1.32 eV without SOC, and 1.036 eV with SOC, which indicates that SOC not only reduces the bandgap values but also causes splitting the of the conduction band minima (CBM). We were able to use SOC to detect a small reduction in the bandgap values for (NH_4_)_2_AgBiBr_6_ and (SbH_4_)_2_AgBiBr_6_ of up to 94 meV and 277 meV, respectively. The band structures for (NH_4_)_2_AgBiBr_6_ and (SbH_4_)_2_AgBiBr_6_ were subsequently calculated using the hybrid model [see Fig. 3(f) and 4(f)]. The calculated indirect bandgap values for (NH_4_)_2_AgBiBr_6_ and (SbH_4_)_2_AgBiBr_6_ are 1.75 eV and 1.44 eV, respectively. When we calculated the bandgaps of PH_4_ and AsH_4_ cations with Cl or Br using the hybrid method (shown in Fig. 5 and 6), they were higher than 2 eV because SOC did not strongly effect the splitting of the CBM, as shown in Table 1. The bandgap values of (NH_4_)_2_AgBiBr_6_ and (SbH_4_)_2_AgBiBr_6_ are closer to the values of CH_3_NH_3_PbX_3_ (X = Cl, Br, I) determined experimentally. We can create an effective semiconductor solar cell material with bandgaps in the region between 1.0 and 1.8 eV. Electrons can be discharged in this bandgap range without generating a significant quantity of heat. Even though the indirect bandgap for double perovskite was recently discovered. While some materials have bandgap values that fall within the specified range, pressure-induced bandgap tuning may be used to achieve bandgap values that are nearly 1.5 eV.

**Fig. 7:**
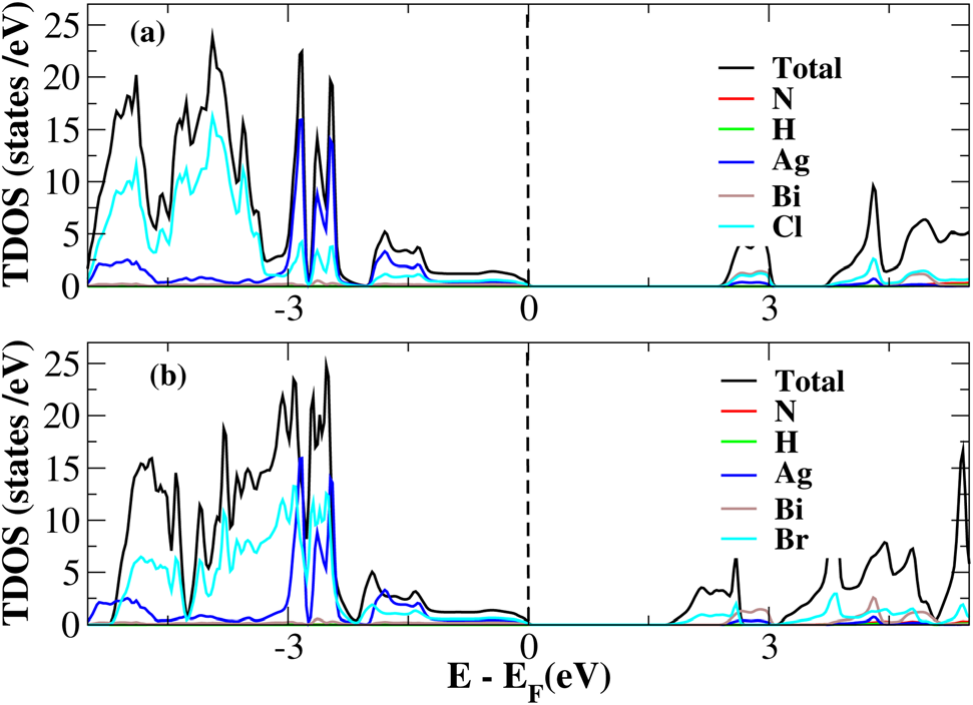
Total DOS of (NH_4_)_2_AgBiY_6_.

**Fig. 8:**
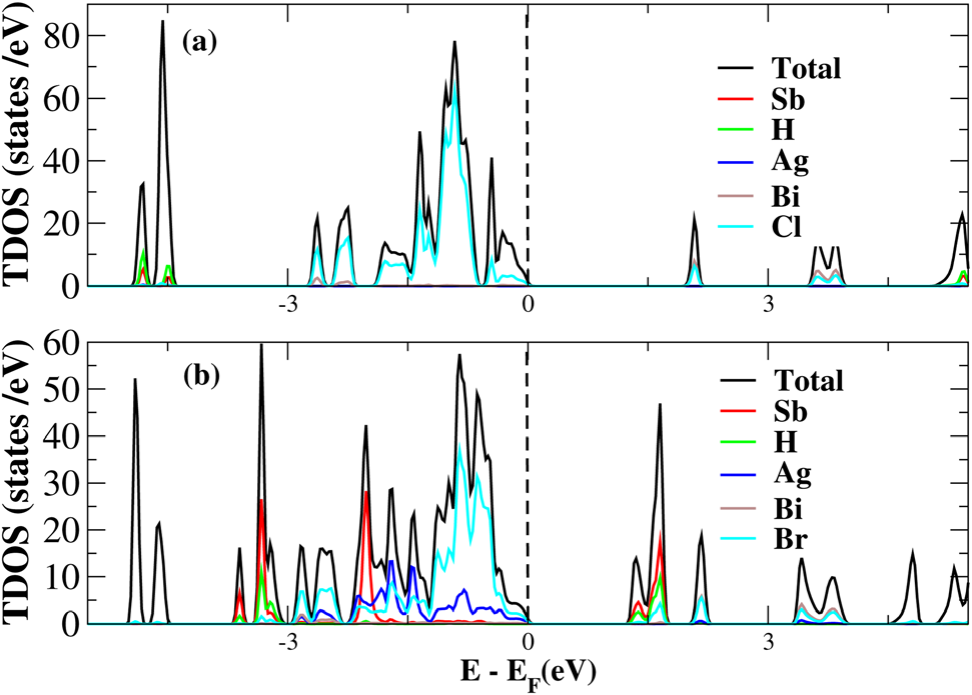
Total DOS of (SbH_4_)_2_AgBiY_6_.

After investigating the band structures, a study of the electrical characteristics followed. The bandgap between the valence band and the conduction band in the Fermi level in these materials causes them to exhibit semiconducting characteristics, as indicated by the plot of the total density of states (TDOS) of (NH_4_)_2_AgBiY_6_ (Y = Cl, Br) and (SbH_4_)_2_AgBiY_6_ (Y = Cl, Br) in Fig. 7 and 8, respectively. A chemical difference serves as the main cause of the indirect bandgap, and the presence of the Ag atom’s 4d state contributes to the compact bandgap in X_2_AgBiY_6_. For the X_2_AgBiY_6_ compounds, the partial density of states (PDOS) comprises the antibonding states of the Ag atom’s 4d orbitals and Br atom’s 4p orbitals, as well as the valence band maxima.

**Fig. 9:**
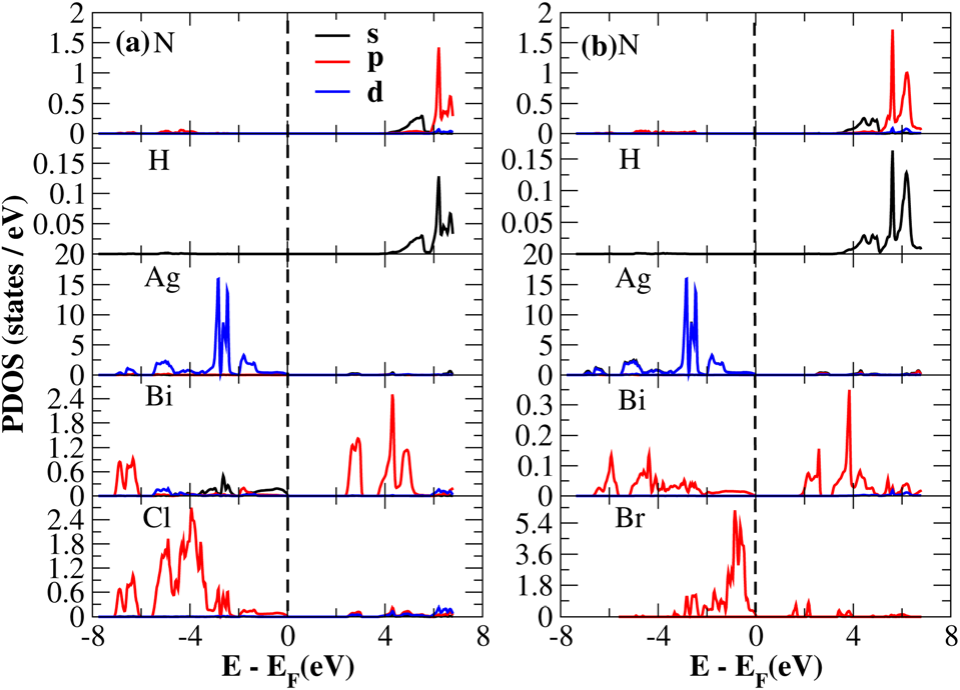
Partial DOS of (NH_4_)_2_AgBiY_6_.

**Fig. 10:**
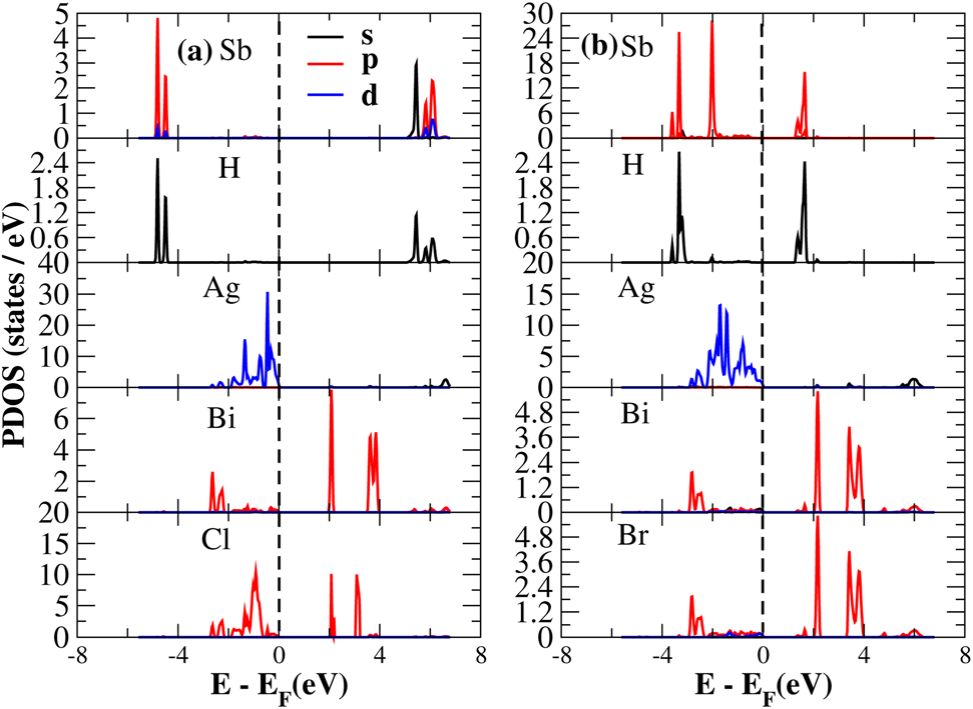
Partial DOS of (SbH_4_)_2_AgBiY_6_.

The PDOS of (NH_4_)_2_AgBiY_6_ (Y = Cl, Br) and (SbH_4_)_2_AgBiY_6_ (Y = Cl, Br) are depicted in Fig. 9 and 10, respectively. The PDOS provides details about how certain atoms contribute to structural stability. For the X_2_AgBiY_6_ compounds, the PDOS comprises the antibonding states of the Ag atom’s 4d orbitals and Br atom’s 4p orbitals as well as the valence band maxima. The 6p-state of Bi and the 4p-state of Cl/Br antibonding states formed the DP compounds in PDOS. This demonstrates how the H-s orbital for material slightly recedes. This demonstrates H-s orbital contribution is deep in the valence band. Important contributions come from the (Ag-d), (Bi-p), and (Cl/Br-p) states, which are depicted in Fig. 9 and 10. Both of these DPs can be used for indirect bandgap engineering in solar cell applications.

**Fig. 11:**
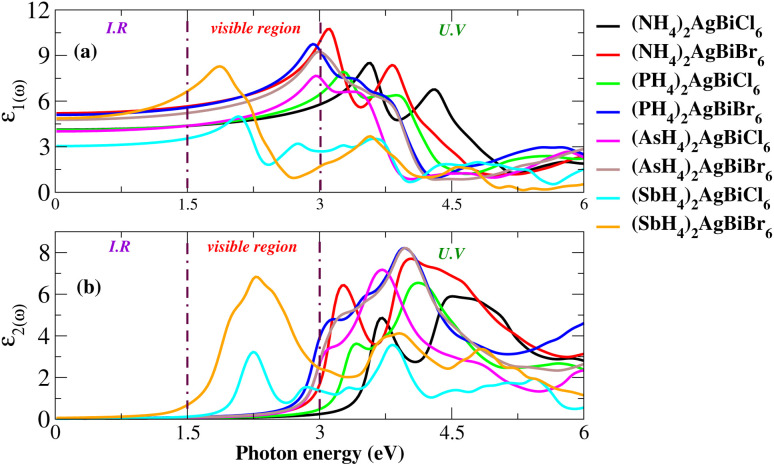
Dielectric function for X_2_AgBiY_6_.

For an understanding of the importance of the predicted materials for solar cell applications, their optical properties were rigorously studied. For solar cell applications, we focused on two compounds, namely (NH_4_)_2_AgBiBr_6_ and (SbH_4_)_2_AgBiBr_6_. Research was undertaken into the optical performance of the transition from the valence band to the conduction band. The interaction of light and objects reveals optical characteristics. For optoelectronic devices, the strength of light absorption and emission depends on the transition between and within bands. Research on the relationship between optoelectronic characteristics and the dielectric function, absorption coefficient, complex refractive-index, and reflectivity *R* is available. The dielectric functions are described by *ε*(*ω*) = *ε*_1_(*ω*) + *iε*_2_(*ω*). Fig. 11(a and b) show the dielectric function plot with an energy range of 0 to 6 eV. The dielectric constant has two parts: the real part, represented by *ε*_1_, and the imaginary part, represented by *ε*_2_. The 0 Hz limit of the *ε*_1_ spectrum exclusively describes the electronic component of the dielectric function, its most significant component. In Fig. 11(a), *ε*_0_ increased from 3 to 5.7. Peaks at 1.75 eV for (SbH_4_)_2_AgBiBr_6_ and 3.2 eV for (NH_4_)_2_AgBiBr_6_ are the highest-energy peaks that have been recorded for these compounds. Thus, according to Penn’s model,^35,36^ the bandgap and static-dielectric constant *ε*_0_ are related to one another. A possible inter-band transition caused by light-energy absorption has been connected to *ε*_2_. This can be seen in Fig. 11(b), where light absorption mostly targets the visible portions of the two predicted molecules. (SbH_4_)_2_AgBiBr_6_’s smaller bandgap polarizes electron transfer in the conductance band and improves the photovoltaic outcomes. An extensive analysis of *ε*_1_ and *ε*_2_ revealed that both (NH_4_)_2_AgBiBr_6_ and (SbH_4_)_2_AgBiBr_6_ were capable of absorbing light with a substantial wavelength amplitude in the energy range of 1 to 4 eV. These combinations should not be ignored as they work well with a variety of solar cell technologies.

**Fig. 12:**
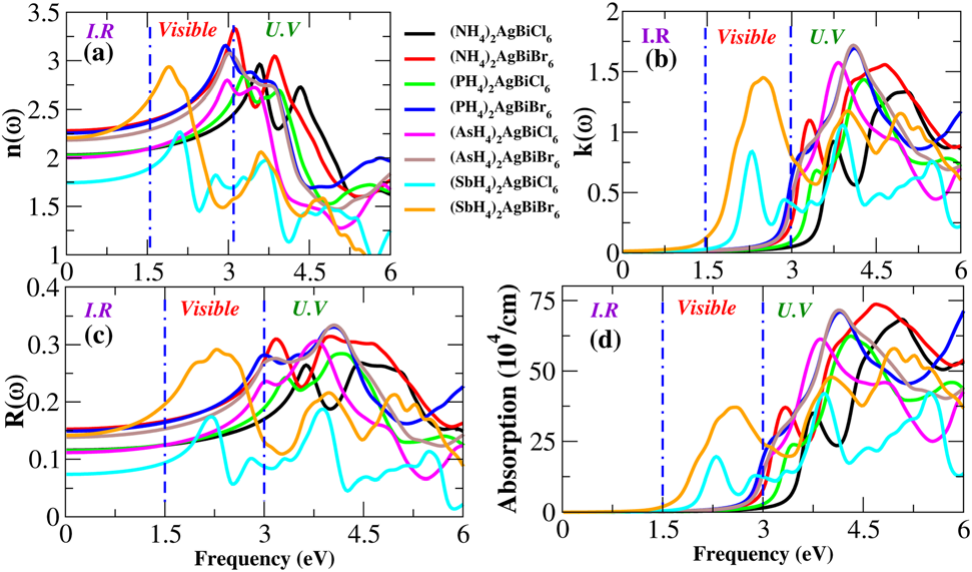
Optical properties (*n*, *k*, *R*, absorption) of X_2_AgBiY_6_.

Light absorption is expressed by the absorption coefficient. In the visible spectrum, absorption weakened in the material under investigation. The obtained value of the absorption coefficient for the calculated compounds is illustrated in Fig. 12(d). The chemical compounds (NH_4_)_2_AgBiBr_6_ and (SbH_4_)_2_AgBiBr_6_ apparently have high absorption coefficients, as indicated by their strong absorption peaks with heights of 37 × 10^4^ cm^−1^ at 2.8 eV and 70 × 10^4^ cm^−1^ at 4.7 eV, respectively. Due to the DFT-based approximation, the effects of *ε*_2_ and *α*(*ω*) have been compared to a lesser overestimation for the absorption coefficient peak.^37,38^ In the case of opto-electronic applications, the refractive index *n*(*ω*) and reflectivity *R*(*ω*) have been important factors that characterize the right kind of material.

For (NH_4_)_2_AgBiBr_6_ and (SbH_4_)_2_AgBiBr_6_, *n*(*ω*) and *R*(*ω*) have been displayed in Fig. 12(a and c). (NH_4_)_2_AgBiBr_6_ and (SbH_4_)_2_AgBiBr_6_ have static refractive index values of 2.4 eV and 2.2 eV, respectively. The peak value is constant since the value of *n*(*ω*) is a copy of *ε*_1_. According to Fig. 12(c), (NH_4_)_2_AgBiBr_6_ and (SbH_4_)_2_AgBiBr_6_ have full reflection *R*(*ω*) values of 0.07 and 0.05, respectively. According to Drude’s model,^39^ those materials light waves in the visible area may be superluminal for both materials. Following a thorough examination of those DP’s optical properties, it was discovered that their significant absorption in the visible to UV spectrum enhances the likelihood that they could be used in solar cell systems. The dissipation of electromagnetic waves in materials is referred to as *K*(*ω*). The trends of *n*(*ω*) and *K*(*ω*) of (NH_4_)_2_AgBiBr_6_ and (SbH_4_)_2_AgBiBr_6_, respectively, are found to be very similar to those of the real and imaginary parts of the dielectric functions, especially where the peaks are located, indicating that the calculated results are in complete compliance with the relationships between the optical parameters (Table 2).

**Table 2:** Optical parameters of (NH_4_)_2_AgBiBr_6_ and (SbH_4_)_2_AgBiBr_6_

Parameters	(NH_4_)_2_AgBiBr_6_	(SbH_4_)_2_AgBiBr_6_
*ε*_1_(0)	3.80	5.7
*n*(0)	2.5	2.2
*R*(0)	0.07	0.05
*k*(0)	4.6	2.7

**Fig. 13:**
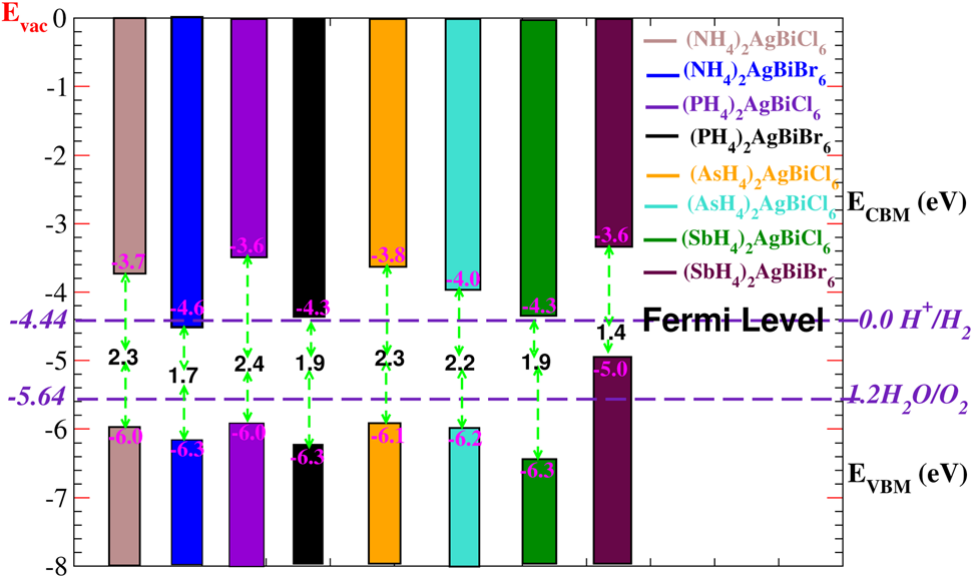
Photocatalytic properties of X_2_AgBiY_6_. The standard oxidation potential *V*_O_2_/H_2_O_ and standard reduction potential *V*_H_2_/H^+^_ for water splitting are −5.67 eV and −4.44 eV (with the latter set as the Fermi level), respectively.

Solar energy can be effectively used by semiconductors with an appropriate bandgap in order to split water molecules and produce hydrogen,^40,41^ therefore, clean renewable energy can be generated using photocatalytic water splitting. The electrons (holes) reduce (oxidize) water during the photocatalytic reaction.^42^ Therefore, the semiconductor’s bandgap needs to be greater than 1.23 eV (Fig. 13). Mulliken electronegativity is used to study photocatalytic water splitting for X_2_AgBiY_6_. *E*_VBM_ = *χ* − *E*_elec_ + 0.5 *E*_g_ and *E*_CBM_ = *E*_VBM_ + *E*_g_.^43,44^ Photocatalytic water splitting for X_2_AgBiY_6_ is studied using Mulliken electronegativity (*χ*), standard electrode potential on the hydrogen scale (*E*_elec_ = 4.5 eV), bandgap (*E*_g_), energies of valence (*E*_VBM_), and conduction (*E*_CBM_) band edge potentials at pH = 0. It is noticeable that the standard oxidation and reduction potentials on the hydrogen scale for photocatalytic water splitting are −4.44 eV (set as the Fermi level) and −5.64 eV, respectively. To determine the band-edge positions of the conduction band (CB) and valence band (VB) with reference to standard oxidation on the hydrogen scale, the Fermi level is set to −4.44 eV.^45^

At pH = 0, the VB and CB are set to 1.23 eV and 0 eV correspondingly, which correspond to −5.67 eV and −4.44 eV.^44^ The VB and CB potentials were estimated for X_2_AgBiY_6_ using the mBJ + (WC-GGA) functional range. The borders of the common redox band satisfy the conditions for water splitting at pH = 0. Out of eight compounds, six compounds show very good photocatalytic properties. (NH_4_)_2_AgBiBr_6_ is not good for reduction purposes and (SbH_4_)_2_AgBiBr_6_ is not good for oxidation purposes. On the other hand, all materials except these two have potentials more positive than the necessary VB potential, indicating good reactions for the oxidation of water. Furthermore, it is evident from Fig. 13 that six materials under study exhibit positions for the *E*_VB_ band edges that are outside of the reduction potentials that are energetically appropriate. Thus, we reach the conclusion that these materials under consideration are viable options for the industrial generation of solar hydrogen.

Effective elastic constants are crucial for appropriate practical applications of a material. The stability of a structure and a material’s reaction to outside forces are both described by the elastic constant. Table 3 lists the computed elastic constants of X_2_AgBiY_6_. Experimental results are not accessible for comparison with our predicted values. Both materials satisfy the *C*_11_ > 0, *C*_44_ > 0, *C*_11_ + 2*C*_12_ > 0, *C*_11_ − *C*_12_ > 0 and *C*_12_ > *B* > *C*_11_ stability criteria for cubic crystals.^46,47^ This demonstrates the elastic stability of both the materials listed above against deformation forces. The elastic constant is being studied in order to calculate the mechanical characteristics using the conventional relations mentioned in Table 3.^48,49^ The material’s plastic deformation in response to applied stress is measured using the shear modulus *G*_H_. The fact that (NH_4_)_2_AgBiBr_6_ has higher Young’s modulus (*Y*) and shear modulus (*G*_H_) values than (Sb_4_)_2_AgBiBr_6_ proves that it is stiffer and offers more resistance to plastic deformation. The *B*/*G* (Pugh ratio) reveals the ductile and brittle behavior of a material.^50^ It is evident from Table 3 that all materials are ductile because their *B*/*G* ratios are greater than 1.75 (the crucial number). The positive or negative values of Cauchy pressure can also be used to indicate a material’s ductile or brittle nature (*C*′′ = *C*_12_ − *C*_44_). The positive value of *C*′′ (in Table 3) also indicates good ductility of (NH_4_)_2_AgBiBr_6_ and (SbH_4_)_2_AgBiBr_6_. The Poisson’s ratio (*v*) shows how resistant a crystal is to compression. Due to the low values of *v*, all materials are determined to be stable in the presence of shear stress. Table 3 lists the values of the anisotropic constant (*A*) for (NH_4_)_2_AgBiBr_6_ and (SbH_4_)_2_AgBiBr_6_. The deviation of *A* from unity can be used to gauge the anisotropy of a material. It is evident from the table that the anisotropic factor is less than unity, and as a result, the properties of the material alter in various crystallographic directions. The shear constant *C* indicates the material’s dynamic stability against tetragonal distortion. For dynamic stability, *C* must be bigger than 0 (positive values).^51^ As shown in Table 3, the fact that all materials have values of *C* that are greater than 0 shows that they are mechanically stable.

**Table 3:** Elastic lattice parameters of X_2_AgBiY_6_

Compound	*C*_11_	*C*_12_	*C*_44_	*G*_V_	*G*_R_	*G*_H_	*Y*	*B*/*G*	*C*′′	*ν*	*A*	*C*′
(NH_4_)_2_AgBiCl_6_	79.19	22.70	18.36	22.31	21.35	21.83	56.76	1.90	4.34	0.27	0.65	28.24
(NH_4_)_2_AgBiBr_6_	84.73	31.04	19.02	22.15	21.53	21.84	58.73	2.25	5.02	0.30	0.70	26.84
(PH_4_)_2_AgBiCl_6_	77.97	28.51	21.98	23.08	23.00	23.04	59.13	1.95	6.52	0.28	0.88	24.72
(PH_4_)_2_AgBiBr_6_	82.98	31.31	24.11	24.80	24.77	24.79	63.58	1.94	7.20	0.27	0.93	25.83
(AsH_4_)_2_AgBiCl_6_	73.37	29.66	21.54	21.66	21.66	21.70	55.88	2.04	8.12	0.28	0.98	21.85
(AsH_4_)_2_AgBiBr_6_	74.83	27.15	21.52	22.45	22.39	24.43	57.37	1.91	5.64	0.27	0.90	23.84
(SbH_4_)_2_AgBiCl_6_	72.21	28.84	20.26	20.83	20.81	20.83	53.85	2.0	8.54	0.29	0.93	21.68
(SbH_4_)_2_AgBiBr_6_	73.38	26.95	19.18	20.79	20.61	20.70	52.30	2.12	7.78	0.28	0.82	23.21

## 4. Conclusions

Double perovskites X_2_Ag^1+^Bi^3+^X_6_ (X = NH_4_, PH_4_, AsH_4_, SbH_4_, and Y = Cl, Br) were investigated using density functional theory. Investigations were done on the structural, electronic, optical, photocatalytic, and elastic properties. These compounds’ bandgaps were computed. From these compounds, only (NH_4_)_2_AgBiBr_6_ and (SbH_4_)_2_AgBiBr_6_ had bandgaps of 1.76 eV and 1.44 eV, respectively. The remaining materials all exhibit bandgap values greater than 2 eV when the mBJ adjustment is applied. In contrast to partial density of states results, which show Cl’s p-orbital bands and Sb’s s-orbital bands, the total density of state (DOS) results present Cl and Br as significant contributors to the valence band maxima (VBM) and Ag as a contributor to the conduction band minima (CBM). We investigated optical characteristics to determine the absorption coefficients of X_2_AgBiY_6_ (NH_4_, PH_4_, AsH_4_, SbH_4_ and Y = Cl, Br) and we learned that, based on the bandgap values and optical absorption, (SbH_4_)_2_AgBiBr_6_ is the best suited option among all other examined compounds for solar cell use. We determined that six compounds are suitable for redox reactions based on their photocatalytic characteristics. The stability, indirect bandgap value, and optical absorption values that are close to MAPI make these perovskites a good choice for lead-free hybrid solar cells. Their stability has been established by their mechanical characteristics.

## Conflicts of interest

The authors declare that they have no known competing financial interests or personal relationships that could have appeared to influence the work reported in this paper.

## Supplementary Material
